# Safety and efficacy of endovascular treatment for progressive large vessel occlusion stroke presenting 24–72 h after onset: a propensity score-matched analysis

**DOI:** 10.3389/fneur.2026.1939194

**Published:** 2026-09-10

**Authors:** Shi-Qian Guo, Yuan-Zhan Guo, Shi-Dun Chen, Zai-Hang Zhang, Shuang Pei, Xing-Chen Qiu, Rong-Yi Liu, Bing Guo, Wan-Li Bao, Jing Zhou, Ning Wang, Jun Sun

**Affiliations:** Department of Neurointervention, Nanyang Central Hospital, Nanyang, Henan, China

**Keywords:** endovascular treatment, progressive stroke, propensity score matching, standard medical therapy, symptomatic intracranial hemorrhage

## Abstract

**Background and purpose:**

To evaluate the efficacy and safety of endovascular treatment (EVT) versus standard medical therapy (SMT) in patients with progressive large vessel occlusion (LVO) presenting 24–72 h after onset.

**Methods:**

We retrospectively analyzed patients with progressive LVO and perfusion mismatch (tissue window) between 24 and 72 h after onset. Propensity score matching (PSM) was performed to balance baseline characteristics. The primary outcome was functional independence (modified Rankin Scale [mRS] 0–2) at 90 days. Secondary outcomes included 90-day mortality and symptomatic intracranial hemorrhage (sICH).

**Results:**

A total of 164 patients were included (EVT, *n* = 48; SMT, *n* = 116). The EVT group showed significantly better 90-day functional outcomes (median mRS: 3 [IQR 2–4] vs. 4 [IQR 3–6]; OR 1.86, 95% CI 1.13–5.25; *p* = 0.018) and higher functional independence rates (43.8% vs. 12.9%; OR 5.24, 95% CI 2.73–10.05; *p* < 0.001) compared with the SMT group. EVT was also associated with reduced severe disability or death (18.8% vs. 47.4%; OR 0.26, 95% CI 0.12–0.56; *p* < 0.001) and lower all-cause mortality (12.5% vs. 37.9%; OR 0.23, 95% CI 0.09–0.51; *p* = 0.001). The incidence of sICH did not differ significantly between groups (10.4% vs. 6.0%; OR 1.81, 95% CI 0.65–5.08; *p* = 0.409). These findings remained consistent after PSM and in sensitivity analyses excluding non-witnessed strokes. Subgroup analysis indicated that good collateral circulation (Tan 2–3) significantly predicted functional independence (73.1% vs. 9.1%; OR 27.14, 95% CI 4.83–152.45; *p* < 0.001).

**Conclusion:**

For progressive LVO patients presenting 24–72 h after onset with favorable perfusion imaging, EVT was associated with improved functional outcomes and lower mortality, without a significant increase in hemorrhage risk. Furthermore, good collateral circulation is a statistically significant factor associated with EVT benefit.

## 1 Introduction

Acute ischemic stroke (AIS) accounts for approximately 80% of all stroke cases and remains a leading cause of global mortality and severe disability (1, 2). Despite substantial advances in reperfusion therapies, up to one-third of patients still experience progressive neurological deterioration within the first week of onset, a condition clinically characterized as progressive stroke (PS) (3, 4). Compared with patients with stable neurological deficits, those with PS are associated with significantly higher rates of morbidity and mortality, representing a critical determinant of poor clinical outcomes.

The pathophysiology of PS is highly complex and heterogeneous, encompassing thrombus propagation, collateral failure, and cerebral edema, among others (5, 6). Notably, there is currently no international consensus on the standardized definition and diagnostic criteria for PS. While landmark trials such as DAWN and DEFUSE 3 have established the efficacy of endovascular treatment (EVT) in the extended window for selected patients, these pivotal studies predominantly enrolled Western populations with stable neurological deficits at presentation (7, 8). More recently, the ANGEL-ASPECT trial provided robust evidence for EVT in Asian patients with large ischemic cores, further validating the benefits of reperfusion therapies in this specific ethnic group (9). However, a critical distinction remains: these trials primarily focused on patients with relatively stable baseline conditions. Currently, there is no international consensus on the standardized definition of PS, and high-quality evidence guiding the management of AIS patients who experience progressive deterioration, particularly those presenting beyond the conventional 24-h window remains scarce. It remains unclear whether the benefits of EVT observed in stable populations can be extrapolated to patients with dynamic neurological worsening.

To address this critical knowledge gap, we conducted this retrospective cohort study to evaluate the clinical outcomes of EVT versus standard medical therapy (SMT) in patients with progressive large vessel occlusion presenting 24 to 72 h after onset. We aimed to elucidate the effectiveness and safety of these two treatment strategies in this specific population, thereby providing preliminary evidence to guide individualized clinical management.

## 2 Materials and methods

### 2.1 Study population

This retrospective cohort study consecutively enrolled patients with progressive acute ischemic stroke and large vessel occlusion (AIS-LVO) presenting in the late window who were admitted to our institution between January 2022 and January 2025. Eligible patients met the following criteria: age between 18 and 80 years; a pre-stroke modified Rankin Scale (mRS) score ≤ 2; a treatment decision (defined as the initiation of endovascular intervention or pharmacological therapy, such as tirofiban) made within 24 to 72 h from symptom onset or last known well (LKW); a baseline National Institutes of Health Stroke Scale (NIHSS) score ≥ 2 with documented neurological deterioration (defined as an increase in the NIHSS score of ≥ 4 points from baseline) within this time window; confirmed intracranial large vessel occlusion on CT angiography (CTA) or MR angiography (MRA); and a significant tissue-window mismatch on CT perfusion (CTP) imaging analyzed with RAPID software (infarct core volume < 70 mL, mismatch ratio ≥ 1.8, and reversible ischemic penumbra volume > 15 mL). The maximum time interval from symptom onset (or LKW) to CTP acquisition was restricted to 72 h to ensure the assessment of salvageable tissue within the late therapeutic window. Exclusion criteria comprised a history of intracranial hemorrhage, active bleeding diathesis, severe cardiac, hepatic, or renal dysfunction, and missing critical imaging or follow-up data.

### 2.2 Treatment methods

#### 2.2.1 Selection and group assignment

For patients presenting within the late window (24–72 h) who satisfied the imaging eligibility criteria, the feasibility of EVT was assessed based on institutional stroke management protocols, imaging findings, comorbidities, and overall clinical presentation. Treatment allocation was then determined through shared decision-making involving the patients’ families. Those who consented to endovascular intervention were assigned to the EVT group, whereas those who opted for conservative management were allocated to the SMT group.

#### 2.2.2 Endovascular procedures

Certified stroke specialists conducted all EVT procedures adhering to the Chinese Guidelines for Endovascular Treatment of Acute Ischemic Stroke and local standard operating procedures. While conscious sedation was preferred, general or composite intravenous anesthesia was administered when airway patency was threatened or agitation was severe. Blood pressure was managed to stay below 180/105 mmHg prior to surgery. Intraoperative irrigation utilized heparinized saline, with systemic heparinization reserved for hypercoagulable patients or anticipated long procedures. After femoral puncture, DSA identified the occlusion site and collateral status. A microcatheter was advanced distal to the clot using a 0.014-inch microwire. A Solitaire AB stent was then deployed, allowed to integrate with the clot for 5 min, and retrieved under negative pressure aspiration. The modified Thrombolysis in Cerebral Infarction (mTICI) scale was used to evaluate reperfusion; multiple passes were performed to achieve a score of ≥2b. For residual stenosis exceeding 70%, rescue angioplasty or stenting was performed alongside intravenous tirofiban administration. Upon completion of the procedure, patients were transitioned to the NICU for a 24-h observation period, during which cranial CT and vascular imaging (MRA/CTA) were obtained. The choice of antiplatelet regimen was determined by stroke etiology: cardioembolic strokes were managed with monotherapy, while large artery atherosclerosis warranted dual antiplatelet therapy (DAPT) following confirmation of no intracranial hemorrhage. Notably, when intraoperative tirofiban was utilized, DAPT was initiated to overlap with the infusion for 4 h before cessation.

#### 2.2.3 Standard medical therapy

The SMT was universally applied based on prevailing guidelines, covering blood pressure and glucose management alongside statins and antiplatelet drugs. The initiation of antiplatelet therapy occurred promptly once intracranial hemorrhage or neurological deterioration was excluded. Treatment strategies were etiology-specific: aspirin combined with clopidogrel was used for large artery atherosclerosis, while a 4-h overlap of DAPT was implemented before discontinuing intraoperative tirofiban. Patients with cardioembolic stroke received initial monotherapy, followed by anticoagulation based on the “1–3–6-12 day” protocol. Derived from the 2018 EHRA practical guide, this rule determines the timing of anticoagulation based on stroke severity (NIHSS score) and the exclusion of hemorrhagic transformation: Day 1 for TIA; Day 3 for mild stroke (NIHSS < 8); Day 6 for moderate stroke (NIHSS 8–15); and Day 12 for severe stroke (NIHSS > 16).

### 2.3 Data collection

Baseline data were systematically collected, encompassing demographic characteristics, medical history (hypertension, diabetes mellitus, atrial fibrillation, etc.), pre-stroke mRS score, NIHSS scores at baseline (admission) and at the time of documented neurological deterioration, and the Alberta Stroke Program Early CT Score (ASPECTS). The site of occlusion in the culprit vessel and collateral circulation status in the affected vascular territory was evaluated using baseline CTA. Collateral circulation was graded according to the Tan score (0–3), with scores of 0–1 defined as poor collaterals and scores of 2–3 as good collaterals. Post-procedural reperfusion in the EVT group was assessed via digital subtraction angiography (DSA), with successful reperfusion defined as a modified Thrombolysis in Cerebral Infarction (mTICI) grade of 2b or 3. For data extraction and quality control, all clinical and imaging data were independently extracted and cross-verified by two neurologists who were uninvolved in the patients’ clinical care. Any discrepancies were resolved through consensus with a third senior researcher. Furthermore, the assessors evaluating the 90-day functional outcomes (mRS scores) were strictly blinded to the treatment allocation and acute management strategies.

### 2.4 Outcomes

The primary efficacy outcome was functional independence at 90 days, defined as a mRS score of 0–2. Secondary outcomes included 90-day all-cause mortality and the incidence of symptomatic intracranial hemorrhage (sICH). sICH was defined according to the ECASS III criteria (9): hemorrhage confirmed on follow-up imaging accompanied by an NIHSS score increase of ≥4 points, after excluding other causes of neurological deterioration such as cerebral edema, seizures, or new ischemic strokes. The imaging surveillance protocol included a routine non-contrast CT at 24 ± 6 h after treatment initiation, with immediate rescanning triggered by any neurological deterioration. The assessment of all outcome measures was performed in a blinded manner. Specifically, the clinical research coordinators responsible for the 90-day follow-up and the evaluation of the mRS were blinded to the patients’ treatment allocation (EVT vs. SMT). Throughout the entire data collection process, we strictly maintained this blinding to minimize any potential assessment bias, particularly for subjective outcomes such as functional status.

### 2.5 Statistical analysis

Statistical analyses were performed using SPSS version 25.0 (IBM Corp., Armonk, NY, United States). Continuous variables with a normal distribution were expressed as mean ± standard deviation (SD) and compared using the independent-samples t-test. Non-normally distributed continuous variables were presented as median (interquartile range [IQR]) and compared using the Wilcoxon rank-sum test. Categorical variables were expressed as frequencies and percentages [*n* (%)] and compared using the Pearson chi-square test or Fisher’s exact test, as appropriate.

To minimize confounding bias, propensity score matching (PSM) was performed at a 1:2 ratio between the EVT and SMT groups, with a caliper width set at 0.02. The propensity score model included demographics (age and sex), medical history (hypertension, diabetes, atrial fibrillation, and premorbid mRS), clinical severity (NIHSS scores at admission and deterioration), and imaging characteristics (ASPECTS, occlusion site, and collaterals). Covariate balance was assessed using standardized mean differences (SMD), with an SMD < 0.10 indicating adequate balance. Binary logistic regression analyses were conducted to evaluate the independent predictive value of EVT for 90-day functional outcomes, mortality, and sICH. Notably, the covariates included in this regression model were identical to those used in the propensity score model. The results are presented as adjusted odds ratios (ORs) with 95% confidence intervals (CIs). Additionally, a sensitivity analysis restricted to patients with witnessed onset was performed to verify the robustness of the findings. Stratified analyses based on collateral circulation status were also conducted within the EVT subgroup to explore the potential modulating effect of collateral compensation on clinical outcomes. Notably, both the sensitivity analysis and the subgroup analyses were conducted based on the per-protocol (PP) population. All statistical tests were two-sided, and a *p* < 0.05 was considered statistically significant.

## 3 Results

### 3.1 Patient selection

**Figure 1:**
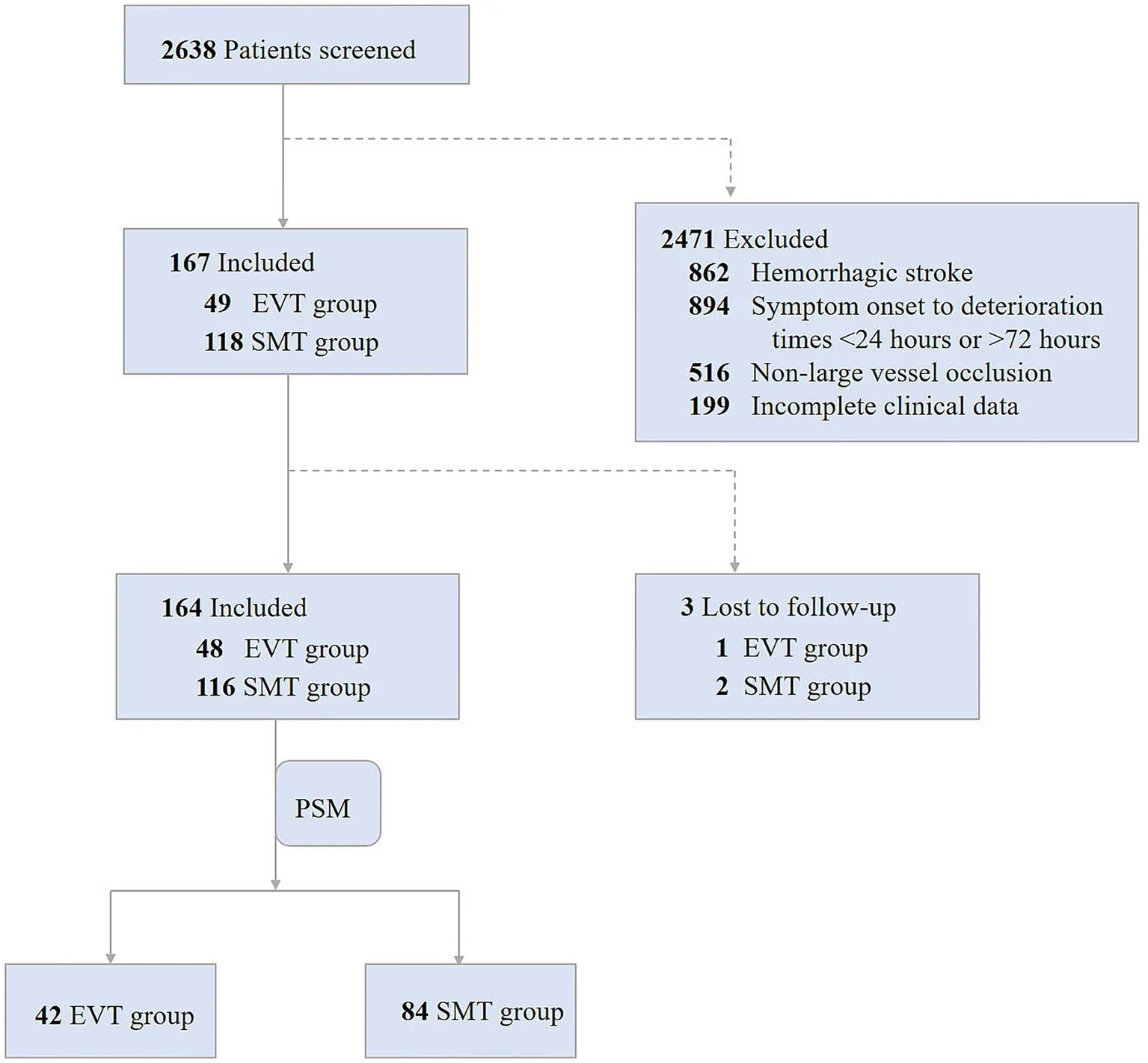
Patient enrollment flowchart.

Between January 2022 and January 2025, a total of 2,638 patients with stroke were admitted to our institution. Following the screening process, 167 patients were ultimately enrolled. The excluded patients comprised those with hemorrhagic stroke (*n* = 862), a time interval from symptom onset to progression and subsequent treatment decision exceeding the 24- to 72-h window (*n* = 894), non-large vessel occlusion (*n* = 516), and missing clinical data (*n* = 199). Of the included patients, 49 were assigned to the EVT group and 118 to the SMT group (Figure 1).

### 3.2 Baseline characteristics

In the initial cohort, patients in the EVT group tended to be slightly older (63 vs. 60 years), had a higher prevalence of hypertension (61.2% vs. 56.7%), and presented with more severe neurological deficits (higher admission NIHSS scores) compared to the SMT group. Additionally, the proportion of patients with good collateral circulation was marginally lower in the EVT group (55.1% vs. 61.0%). However, none of these differences, including time window metrics, reached statistical significance (all *p* > 0.05). Notably, 3 patients in the initial cohort were lost to follow-up (1 in the EVT group and 2 in the SMT group) and were excluded from subsequent analyses. Ultimately, a total of 164 patients were included in the PSM pool. Following matching, 126 patients were included in the primary analysis (42 in the EVT group and 84 in the SMT group). Post-matching baseline characteristics demonstrated excellent balance between the two groups. All measured covariates were well-balanced, with SMD < 0.10, indicating that the matching procedure effectively minimized selection bias (Table 1).

**Table 1:** Baseline characteristics.

Variables	Before PSM			After PSM			SMD
	EVT (*n* = 49)	SMT (*n* = 118)	*P*	EVT (*n* = 42)	SMT (*n* = 84)	*P*	
Age (year), median (IQR)	63 (55–71)	60 (53–69)	0.269	63 (55–69)	60 (53–67)	0.372	0.055
Sex (male), *n* (%)	29 (59.2)	74 (62.7)	0.669	25 (59.5)	53 (63.1)	0.696	0.024
High-risk factors, *n* (%)							
Hypertension	30 (61.2)	67 (56.7)	0.596	27 (64.3)	47 (56.0)	0.370	0.083
Diabetes	20 (40.8)	62 (52.5)	0.168	17 (40.5)	42 (50.0)	0.312	0.062
Coronary artery disease	10 (20.4)	38 (32.2)	0.125	9 (21.4)	24 (28.6)	0.390	0.056
Atrial fibrillation	6 (12.2)	7 (5.9)	0.165	5 (11.9)	6 (7.1)	0.372	0.067
Smoking	20 (40.8)	45 (38.1)	0.746	18 (42.9)	35 (41.7)	0.900	0.022
Alcohol consumption	12 (24.5)	23 (19.5)	0.470	11 (26.2)	19 (22.6)	0.656	0.037
Medication before admission, *n* (%)							
Anticoagulant, *n* (%)	3 (6.1)	11 (9.3)	0.497	3 (7.1)	10 (11.9)	0.406	0.040
Antiplatelet, *n* (%)	13 (26.5)	40 (33.9)	0.351	12 (28.6)	31 (36.9)	0.352	0.016
NIHSS score at admission, median (IQR)	8 (6–12)	7 (5–12)	0.293	8 (6–11)	7 (5–11)	0.314	0.033
ASPECTS score at admission, median (IQR)	8 (7–9)	9 (8–9)	0.447	8 (7–9)	8 (7–9)	0.556	0.021
NIHSS score at neurological deterioration, median (IQR)	12 (9–15)	11 (7–14)	0.582	12 (9–14)	11 (7–14)	0.493	0.019
Locations of infarction, *n* (%)							
ICA	18 (36.7)	49 (41.5)	0.564	16 (38.1)	33 (39.3)	0.898	0.013
MCA	27 (55.1)	61 (51.7)	0.688	22 (52.4)	43 (51.2)	0.900	0.011
ICA and MCA	4 (8.2)	8 (6.8)	0.752	4 (9.5)	8 (9.5)	1.000	0.000
Collateral status (0–3), *n* (%)							
Tan 2–3	27 (55.1)	72 (61.0)	0.478	23 (54.8)	50 (59.5)	0.610	0.042
Tan 0–1	22 (44.9)	46 (39.0)	0.478	19 (45.2)	34 (40.5)	0.610	0.042
Time from symptom onset or LKW to, median (IQR)							
Arrive at the hospital	13 (7–22)	11 (6–23)	0.349	13 (7–21)	12 (6–22)	0.374	0.064
Neurological deterioration	30 (26–63)	31 (21–67)	0.148	30 (26–62)	31 (26–65)	0.552	0.068
Treatment with EVT or SMT	34 (28–69)	33 (28–70)	0.457	34 (28–68)	33 (28–69)	0.639	0.037
EVT modalities							
Mechanical thrombectomy alone	34	NA	NA	28	NA	NA	NA
Combined with balloon angioplasty	8	NA	NA	8	NA	NA	NA
Combined with stenting	7	NA	NA	6	NA	NA	NA
Reperfusion Status							
Unsuccessful (mTICI 0–2a)	4 (8.2)	NA	NA	2 (4.8)	NA	NA	NA
Successful (mTICI 2b-3)	45 (91.8)	NA	NA	40 (95.2)	NA	NA	NA

EVT, endovascular treatment; SMT, standard medical therapy; PSM, propensity score matching; SMD, standardized mean differences; IQR, Interquartile range; mRS, modified Rankin Scale; ASPECTS, Alberta Stroke Program Early CT Score; NIHSS, National Institutes of Health Stroke Scale; ICA, internal carotid artery; MCA, middle cerebral artery; LKW, last known well; mTICI, modified Thrombolysis in Cerebral Infarction.

### 3.3 Clinical outcomes

**Figure 2:**
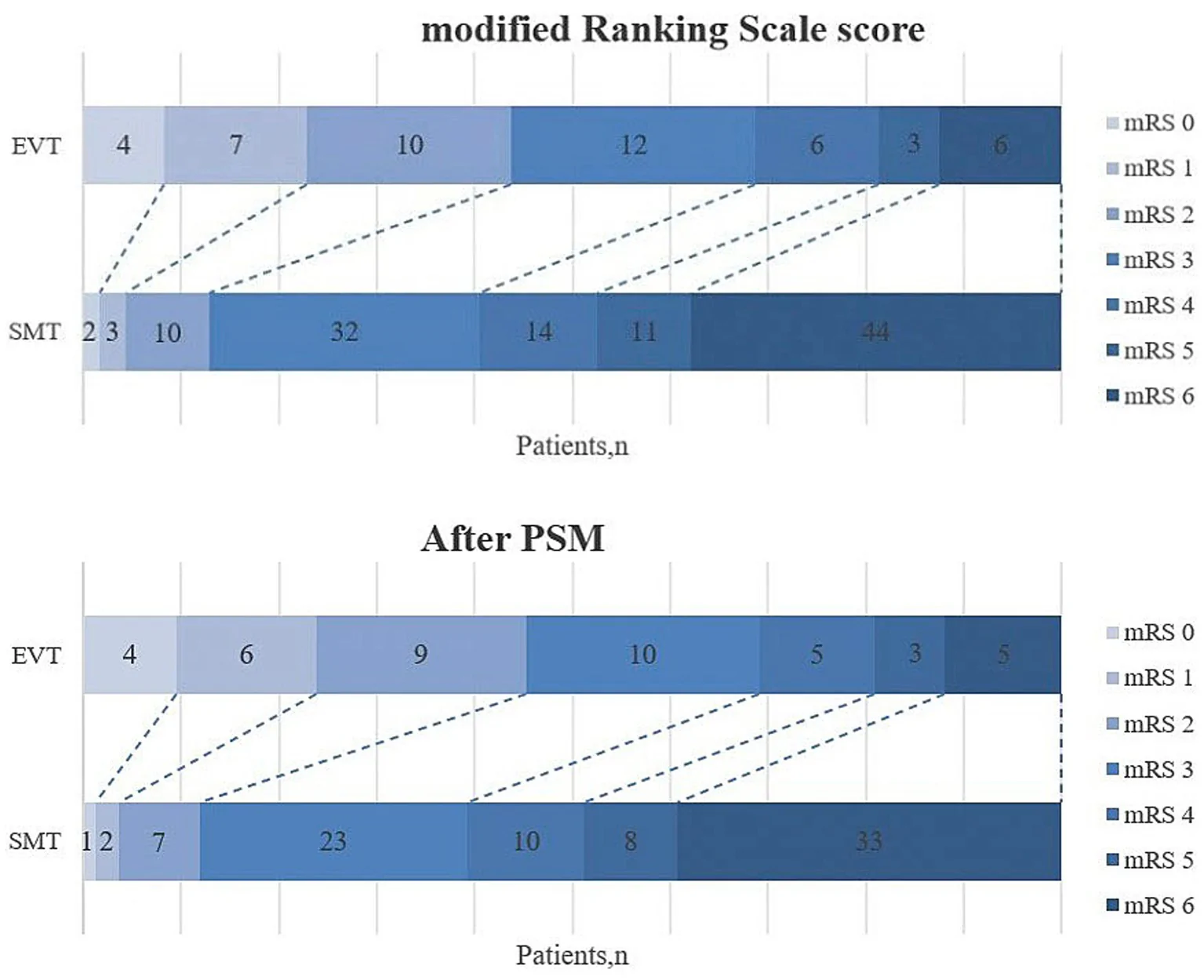
Distribution of mRS scores in the original cohort and after PSM: *p* < 0.05.

Compared with the SMT group, patients in the EVT group achieved significantly better 90-day mRS scores [median 3 (IQR, 2–4) vs. 4 (IQR, 3–6); OR = 1.86, 95% CI 1.13–5.25, *p* = 0.018; Figure 2]. The proportion of patients achieving favorable functional outcomes (mRS ≤ 2) was significantly higher in the EVT group (43.8% vs. 12.9%; OR = 5.24, 95% CI 2.73–10.05, *p* < 0.001), whereas the proportion of patients experiencing severe disability or death (mRS ≥ 5) was significantly lower (18.8% vs. 47.4%; OR = 0.26, 95% CI 0.12–0.56, *p* < 0.001).

Regarding safety, the 90-day all-cause mortality rate was significantly lower in the EVT group than in the SMT group (12.5% vs. 37.9%; OR = 0.23, 95% CI 0.09–0.51, *p* = 0.001). Although the incidence of sICH was numerically higher in the EVT group (10.4% vs. 6.0%), the difference did not reach statistical significance (OR = 1.81, 95% CI 0.65–5.08, *p* = 0.409). All outcome data are detailed in Table 2.

**Table 2:** Outcome measures.

Outcomes	EVT (*n* = 48)	SMT (*n* = 116)	OR (95% CI)	*P*	adjust OR (95% CI)	*P*
mRS at 90 days	3 (2–4)	4 (3–6)	1.86 (1.13–5.25)	0.018	1.66 (1.11–4.95)	0.012
mRS ≤ 2	21 (43.8)	15 (12.9)	5.24 (2.73–10.05)	<0.001	5.00 (2.21–11.18)	0.001
mRS ≥ 5	9 (18.8)	55 (47.4)	0.26 (0.12–0.56)	<0.001	0.32 (0.14–0.48)	0.001
90-day mortality	6 (12.5)	44 (37.9)	0.23 (0.09–0.51)	0.001	0.220(0.08–0.62)	0.006
sICH	5 (10.4)	7 (6.0)	1.81 (0.65–5.08)	0.409	1.66 (0.55–5.04)	0.625

OR, odds ratio; CI, confidence interval; mRS, modified Rankin Scale; sICH, symptomatic intracranial hemorrhage.

### 3.4 Sensitivity analysis

There were 33 patients with an unwitnessed onset in this study. Following their exclusion, the primary findings remained highly consistent with the overall analysis. The 90-day mRS scores in the EVT group continued to be significantly superior to those in the SMT group [median 3 (IQR, 2–4) vs. 4 (IQR, 3–6); OR = 1.64, 95% CI 1.04–4.94, *p* = 0.032]. Functional independence (mRS ≤ 2) was achieved by a significantly higher proportion of patients in the EVT group (39.4% vs. 12.9%; OR = 4.40, 95% CI 1.92–9.19, *p* < 0.001), while the risk of severe disability or death (mRS ≥ 5) was significantly lower (18.4% vs. 48.3%; OR = 0.24, 95% CI 0.09–0.62, *p* = 0.002). Furthermore, the 90-day mortality rate remained significantly lower in the EVT group (10.5% vs. 37.6%; OR = 0.20, 95% CI 0.06–0.61, *p* = 0.002), with no significant difference in sICH incidence between the two groups (10.5% vs. 6.5%; OR = 1.71, 95% CI 0.44–6.61, *p* = 0.741). Detailed data are presented in Table 3.

**Table 3:** Sensitivity analysis of outcome measures.

Outcomes	EVT (*n* = 38)	SMT (*n* = 93)	OR (95% CI)	*P*
mRS at 90 days	3 (2–4)	4 (3–6)	1.64 (1.04–4.94)	0.032
mRS ≤ 2	15 (39.4)	12 (12.9)	4.40 (1.92–9.19)	<0.001
mRS ≥ 5	7 (18.4)	45 (48.3)	0.24 (0.09–0.62)	0.002
90-day mortality	4 (10.5)	35 (37.6)	0.20 (0.06–0.61)	0.002
sICH	4 (10.5)	6 (6.5)	1.71 (0.44–6.61)	0.741

### 3.5 Subgroup analysis

Within the EVT group, collateral circulation status significantly influenced clinical outcomes. Compared with patients with poor collaterals (Tan score 0–1), those with good collaterals (Tan score 2–3) had significantly lower 90-day mRS scores [median 3 (IQR, 2–4) vs. 4 (IQR, 3–5); OR = 1.60, 95% CI 1.08–2.32, *p* = 0.003] and a substantially higher rate of functional independence (mRS ≤ 2) (73.1% vs. 9.1%; OR = 27.14, 95% CI 4.83–152.45, *p* < 0.001). Regarding safety, the 90-day mortality rate was numerically lower in the good collaterals group, although the difference did not reach statistical significance (3.8% vs. 22.7%; OR = 0.14, 95% CI 0.01–1.35, *p* = 0.099). No significant difference was observed in sICH incidence between the two groups (7.7% vs. 13.6%; OR = 0.56, 95% CI 0.08–3.74, *p* = 1.000). Detailed data are summarized in Table 4.

**Table 4:** Subgroup analysis of outcome measures in EVT group.

Outcomes	EVT group		OR (95% CI)	*P*
	Tan 2–3	Tan 0–1		
mRS at 90 days	3 (2–4)	4 (3–5)	1.60 (1.08–2.32)	0.003
mRS ≤ 2	19 (73.1)	2 (9.1)	27.14 (4.83–152.45)	<0.001
90-day mortality	1 (3.8)	5 (22.7)	0.14 (0.01–1.35)	0.099
sICH	2 (7.7)	3 (13.6)	0.56 (0.08–3.74)	1.000

## 4 Discussion

In this propensity score-matched analysis of patients with progressive acute ischemic stroke and large vessel occlusion presenting in the late window of 24 to 72 h, EVT was associated with significantly improved 90-day functional outcomes and reduced mortality, without increasing the risk of symptomatic intracranial hemorrhage. These findings underscore the importance of shifting focus from rigid time-based limitations to individualized reperfusion strategies guided by the tissue window and dynamic clinical evolution.

Neurological deterioration is an independent predictor of poor prognosis, occurring in approximately 25 to 40% of patients during the early phase of stroke onset (10–12). However, current guidelines lack high-level recommendations for managing progressive stroke beyond the 24-h mark. While landmark trials such as DAWN and DEFUSE-3 successfully extended the EVT window to 24 h, they did not incorporate “dynamic clinical progression” as a core selection criterion (8, 13). Emerging evidence suggests that progressive stroke reflects the dynamic decompensation of collateral circulation, implying that salvageable penumbra may still persist in these patients even beyond the conventional time window (14, 15). Our study addresses this gap by integrating “neurological deterioration” with “imaging tissue-window mismatch” to precisely identify a subpopulation that retains reperfusion potential within the 24- to 72-h late window. Consistent with this pathophysiology-driven approach, patients treated with EVT showed a significantly higher proportion of favorable functional outcomes at 90 days compared to the SMT group (43.8% vs. 12.9%). These findings align with the trends observed in the SELECT2, RESCUE-Japan LIMIT, and ANGEL-ASPECT trials, further reinforcing that individualized decision-making based on dynamic pathophysiological evolution can yield substantial clinical benefits (9, 16, 17).

The accurate determination of the “LKW” time is a critical determinant of treatment decision reliability in studies involving the extended time window (18). In cases of unwitnessed stroke onset, LKW often relies on caregiver recall or estimation, which introduces potential recall bias and may compromise the validity of the 24- to 72-h window definition. To address this, our sensitivity analysis restricted to patients with witnessed onset demonstrated that the EVT group maintained highly consistent benefits in functional independence (39.4% vs. 12.9%) and mortality (10.5% vs. 37.6%) compared with the primary analysis. These findings confirm that the clinical benefits of EVT are not artifacts of LKW estimation errors but genuinely reflect the pathophysiological advantages of reperfusion in progressive stroke patients beyond the conventional window. This further validates the robustness of “tissue-window” based decision-making, even in scenarios with unwitnessed stroke onset.

The compensatory capacity of collateral circulation is generally considered an important factor influencing infarct volume expansion and clinical outcomes in acute ischemic stroke (19, 20). Our subgroup analysis suggests that there may be an interaction between collateral status and the clinical efficacy of EVT, indicating that the magnitude of benefit from reperfusion therapy may be closely related to the underlying collateral compensation of the brain tissue. Specifically, among patients treated with EVT, those with robust collaterals (Tan 2–3) tended to achieve a higher rate of functional independence at 90 days compared to those with poor collaterals (73.1% vs. 9.1%). From a pathophysiological perspective, this clinical disparity may reflect the hemodynamic role of collateral circulation in sustaining the ischemic penumbra. Robust collateral flow may help maintain cerebral perfusion pressure in the ischemic region, potentially delaying the transition of ischemic tissue into irreversible infarct core, thereby possibly extending the therapeutic time window and contributing to the preservation of microvascular integrity. This relative stability of the microenvironment may not only facilitate tissue salvage but also potentially reduce the risk of blood–brain barrier disruption. Conversely, collateral failure may be accompanied by infarct core expansion and microcirculatory impairment. In such a pathological milieu, delayed recanalization may not fully salvage the ischemic tissue, and due to potential compromise of vascular wall integrity, it may increase the risk of futile reperfusion or hemorrhagic transformation. We acknowledge that this subgroup analysis was limited by a small sample size, resulting in wide confidence intervals for the good collateral group. Future prospective studies are warranted to validate the predictive value of collateral status and to explore the potential synergistic benefits of collateral-augmenting strategies.

Safety remains a paramount concern in reperfusion therapy for the extended time window. In our study, the incidence of sICH in the EVT group was 10.4%. Although numerically higher than that in the SMT group (6.0%), this difference did not reach statistical significance and was notably lower than the hemorrhage rates reported in previous extended window trials (21–23). This favorable safety profile is likely attributable to our stringent imaging selection criteria, specifically, an initial infarct core volume < 70 mL and a hypoperfusion-to-infarct core ratio ≥ 1.8. These thresholds effectively excluded patients with large ischemic cores who are at elevated risk for reperfusion injury and hemorrhagic transformation. Concurrently, the 90-day all-cause mortality was significantly lower in the EVT group compared with the SMT group (12.5% vs. 37.9%), indicating that the survival benefit conferred by reperfusion substantially outweighs the potential hemorrhagic risks. Taken together with the robust findings from our sensitivity analysis, these safety data offer critical guidance for risk–benefit assessment in the extended window: for patients meeting strict criteria for clinical progression and imaging mismatch, endovascular intervention should not be withheld solely due to elapsed time or uncertainties regarding the LKW.

This study is subject to several limitations. First, the retrospective, non-randomized design introduces inherent selection bias. Although we employed propensity score matching and sensitivity analyses to mitigate this, the potential influence of unmeasured confounders cannot be entirely excluded. Second, this was a single-center study with a relatively modest sample size. Due to the limited sample size, all results of this study should be interpreted with caution. Notably, the low incidence of functional independence within the poor collaterals subgroup resulted in wide confidence intervals for the odds ratios; therefore, these subgroup findings should be interpreted with particular caution and require validation in larger cohorts. Third, specific treatment protocols, particularly the pharmacological regimens in the SMT group reflect institutional practices, which may limit the generalizability of our findings to broader clinical settings. Finally, the follow-up was restricted to 90 days, precluding the assessment of long-term functional durability and quality of life. Future multicenter, prospective, randomized controlled trials are needed to establish standardized protocols for endovascular treatment in progressive stroke beyond the conventional window.

## 5 Conclusion

In patients with acute large vessel occlusion presenting in the late 24- to 72-h window characterized by progressive neurological deterioration and imaging evidence of salvageable tissue, EVT was associated with superior functional outcomes and reduced mortality compared with SMT, without increasing the risk of severe intracranial hemorrhage. These benefits remained consistent even after excluding patients with an unwitnessed onset. Furthermore, collateral circulation status emerged as a statistically significant factor associated with treatment response; however, this finding should be interpreted with caution due to the small sample size and limited statistical power. These findings suggest that, with rigorous patient selection, EVT may represent a viable therapeutic strategy for progressive stroke beyond the conventional window, providing a rationale for incorporating these findings into future clinical guidelines.

## Data Availability

The data analyzed in this study is subject to the following licenses/restrictions: the original contributions presented in the study are included in the article, further inquiries can be directed to the corresponding author. Requests to access these datasets should be directed to Sunjun198686@163.com.

## Glossary

EVT: endovascular treatment
SMT: standard medical therapy
LVO: large vessel occlusion
PSM: propensity score matching
mRS: modified Rankin Scale
sICH: symptomatic intracranial hemorrhage
AIS: acute ischemic stroke
PS: progressive stroke
AIS-LVO: acute ischemic stroke with large vessel occlusion
LKW: last known well
CTA: CT angiography
MRA: MR angiography
CTP: CT perfusion
ASPECTS: Alberta Stroke Program Early CT Score
DSA: digital subtraction angiography
mTICI: modified Thrombolysis in Cerebral Infarction
IQR: interquartile range
SMD: standardized mean differences
OR: odds ratios
CI: confidence intervals
NIHSS: National Institutes of Health Stroke Scale
SD: standard deviation
